# Statistical design and analysis plan for an impact evaluation of an HIV treatment and prevention intervention for female sex workers in Zimbabwe: a study protocol for a cluster randomised controlled trial

**DOI:** 10.1186/s13063-015-1095-1

**Published:** 2016-01-05

**Authors:** James R. Hargreaves, Elizabeth Fearon, Calum Davey, Andrew Phillips, Valentina Cambiano, Frances M. Cowan

**Affiliations:** Centre for Evaluation Department for Social and Environmental Health Research, Faculty of Public Health and Policy, London School of Hygiene and Tropical Medicine, 15-17 Tavistock Place, London, WC1H 9SH UK; Research Department of Infection and Population Health, Institute of Epidemiology and Health Care, Faculty of Population Health Sciences, University College London, Gower Street, London, WC1E 6BT UK; Centre for Sexual Health & HIV/AIDS Research (CeSHHAR) Zimbabwe, 9 Monmouth Road Avondale West, Harare, Zimbabwe

**Keywords:** Cluster randomised control trial, respondent driven sampling, pragmatic trials, effectiveness, sex workers, hidden populations, HIV/AIDS

## Abstract

**Background:**

Pragmatic cluster-randomised trials should seek to make unbiased estimates of effect and be reported according to CONSORT principles, and the study population should be representative of the target population. This is challenging when conducting trials amongst ‘hidden’ populations without a sample frame. We describe a pair-matched cluster-randomised trial of a combination HIV-prevention intervention to reduce the proportion of female sex workers (FSW) with a detectable HIV viral load in Zimbabwe, recruiting via respondent driven sampling (RDS).

**Methods:**

We will cross-sectionally survey approximately 200 FSW at baseline and at endline to characterise each of 14 sites. RDS is a variant of chain referral sampling and has been adapted to approximate random sampling. Primary analysis will use the ‘RDS-2’ method to estimate cluster summaries and will adapt Hayes and Moulton’s ‘2-step’ method to adjust effect estimates for individual-level confounders and further adjust for cluster baseline prevalence. We will adapt CONSORT to accommodate RDS. In the absence of observable refusal rates, we will compare the recruitment process between matched pairs. We will need to investigate whether cluster-specific recruitment or the intervention itself affects the accuracy of the RDS estimation process, potentially causing differential biases. To do this, we will calculate RDS-diagnostic statistics for each cluster at each time point and compare these statistics within matched pairs and time points. Sensitivity analyses will assess the impact of potential biases arising from assumptions made by the RDS-2 estimation.

**Discussion:**

We are not aware of any other completed pragmatic cluster RCTs that are recruiting participants using RDS. Our statistical design and analysis approach seeks to transparently document participant recruitment and allow an assessment of the representativeness of the study to the target population, a key aspect of pragmatic trials. The challenges we have faced in the design of this trial are likely to be shared in other contexts aiming to serve the needs of legally and/or socially marginalised populations for which no sampling frame exists and especially when the social networks of participants are both the target of intervention and the means of recruitment.

The trial was registered at Pan African Clinical Trials Registry (PACTR201312000722390) on 9 December 2013.

## Background

HIV/AIDS remains a public health problem of unprecedented scale in many low- and middle-income countries, particularly in southern Africa. Incidence has peaked in many countries but remains unacceptably high at an estimated 1.5 million across sub-Saharan Africa in 2013. Similarly, treatment has expanded, but a wide gap exists in treatment: only 29 % of those who are HIV positive in the region are estimated to be virally suppressed, and 1.1 million HIV-related deaths occurred in sub-Saharan Africa in 2013 [1]. Increasingly, the challenge is in finding models of intervention that can be successfully implemented at scale to ensure universal, equitable access to effective treatment and prevention technologies.

In many sub-Saharan African countries, female sex workers (FSW) and their clients remain at very high risk of acquiring HIV [2], and may have reduced access to treatment [2]. In Zimbabwe, data from 2009 to 2013 suggest greater than 10 % annual HIV incidence among FSW [3], that only 67 % are aware of their HIV status and that 49.5 % of all FSW testing positive for HIV have a viral load < 1000 copies/ml (Cowan F, et al. HIV care cascade among female sex workers in Zimbabwe: baseline results of the SAPPH-IRe Trial, submitted). While guidelines for interventions for FSW exist [4], few impact evaluations have addressed how effective such approaches are in practice.

Pragmatic trials seek to estimate population-level intervention or programme effectiveness on target groups under real-life delivery conditions. This leads to a focus in pragmatic trials on *less* researcher-led control of intervention delivery, but a *stronger* requirement to measure fidelity of intervention implementation [5]. Pragmatic trials also seek to maximise the representativeness of the research sample to the source population. These requirements of the pragmatic trials raise particular challenges for their design and analysis, especially where the target population is hidden or marginalised, as is the case for FSW in southern Africa.

The SAPPH-IRe trial in Zimbabwe (‘Sisters Antiretroviral Programme for Prevention of HIV – an Integrated Response’) was initiated in April 2014. The aim of the trial is to estimate the effect of a combination intervention package on the proportion of Zimbabwean FSW who have an HIV viral load ≥ 1000 copies/ml (approximately reflecting whether a person is infectious). The trial is designed as a pragmatic cluster randomised trial with several novel features including recruitment via respondent-driven sampling (RDS). RDS is a variant of chain referral sampling, for which a model of sampling probabilities is applied and used to weight the data to approximate random sampling. This paper outlines the design of the trial, and reports our analysis plan designed to reflect CONSORT principles.

## Interventions

Since 2009, the national ‘Sisters’ programme has provided targeted HIV services for FSW across all regions of Zimbabwe in line with WHO guidelines [4]. This set of services is the usual standard of care available to comparison communities in the SAPPH-IRe trial. The program provides free condoms and contraception, HIV testing and counselling, syndromic management of sexually transmitted infections, and legal advice supported by a network of peer educators. New standard-of-care services being introduced over the trial period include providing long-acting reversible contraception, community mobilisation and real-time electronic data collection. The ‘Sisters’ programme is run by programme staff through dedicated drop-in centres based at primary care clinics at FSW hotspots around the country. Women who require HIV care and/or antiretroviral treatment (ART) are referred to government services.

In the SAPPH-IRe intervention arm, we will enhance access to ART for HIV prevention and treatment for FSW. We are using enhanced community mobilisation to foster an empowering environment and increase uptake of HIV testing, prevention and treatment services. Targeted activities actively engage women in prevention and treatment by (1) raising awareness of the benefits and availability of ARV drugs for treatment and also for pre-exposure prophylaxis (PrEP); (2) strengthening networks of support to encourage health-promoting behaviour, including the promotion of antiretroviral adherence; and (3) building leadership skills among FSW. For women who access services and test HIV-negative, we are implementing a programme of activities designed to increase repeat HIV testing, including with the use of SMS messaging reminders. On the supply side we enhance on-site clinical services to make ARV for treatment and prevention more readily available to FSW [6]. We include an offer of PrEP for HIV-negative FSW, on-site initiation of ART in line with internationally accepted guidelines for those who have tested HIV-positive, and clinical and social support services delivered by clinical staff within this package [7].

## Methods/Design

### Design

**Fig. 1:**
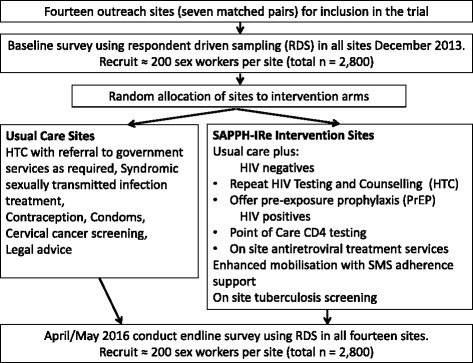
Summary of trial design

The trial is a matched, cluster randomised controlled trial, Fig. 1. Trial sites have been purposively selected to be reflective of a range of settings, they are of adequate size to ensure participation of between 85 and 300 new FSW annually, and they are located at geographic spacing sufficient to ensure that the risk of contamination/spill-over of intervention effect between study clusters through FSW mobility and migration is minimised. A cluster was defined as the FSW population working in the geographic location around a government health clinic where dedicated FSW services are being delivered.

### Matching and randomisation

Trial sites were pair-matched on the basis of the setting (for example, town, growth point and colliery/army base) and whether the site had been providing dedicated FSW services since 2009/10 or was a newer outreach site. We conducted a public randomisation on 31 January 2014 and invited a number of stakeholders, including national and provincial representatives of Ministry of Health and Child Care National AIDS Council and the trial community advisory board. The randomization was performed by inviting non-investigator attendees to draw from a bag the name of one of the sites from each pair. The draw for each pair was performed by a different stakeholder. The first names that were drawn were designated to group 1, and the names of sites left in the bag, to group 2. Finally a coin was first inspected, and then through a toss of this coin, the sites in “group 2” were allocated to the enhanced intervention arm and those in “group 1” to the usual care arm.

### Primary and secondary endpoints

The trial endpoint is the proportion of all FSW who have an HIV viral load ≥ 1000 copies/ml (approximately reflecting whether a person is infectious). This has been assessed at baseline December 2013 and will be assessed again at endline in April/May 2016. The FSW will be recruited using RDS, as described below.

### Sample size calculations

**Fig. 2:**
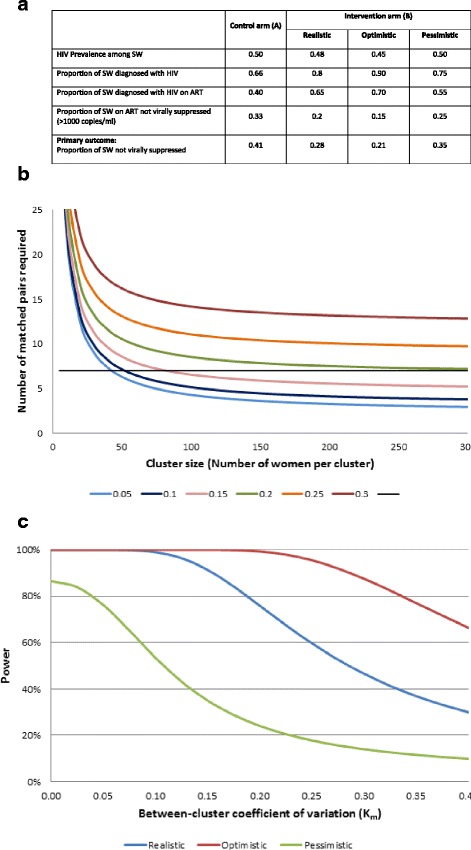
Sample size considerations for the SAPPH-IRe Trial. Notes: (**a**) We estimated that in the comparison arm 41 % of SW will have a detectable viral load at 24 months. The breakdown of hypothesised effect of the intervention on HIV prevalence, the proportion of HIV infected women who are diagnosed, the proportion of diagnosed women who are on ART, and the proportion of women on ART with detectable viral load is shown in Table 2. We hypothesize that with realistic estimates of the size of the potential effect of our intervention on improving knowledge of HIV status among HIV-infected SW, decreasing time to treatment initiation and improving adherence, in the enhanced intervention arm (Arm B) 28 % of SW should be expected to have detectable viral load at the time of the final RDS survey. We also show more ‘optimistic’ and ‘pessimistic’ scenarios. (**b**) Number of matched paired of SW clinics (clusters) and number of women per site required to detect a reduction in proportion of women not virally suppressed (not on ART or virologically failing) from 41 to 28 % (80 % power, 5 % level of significance) for various values of the between-cluster coefficient of variation (km) in the proportion not virally suppressed within matched pairs. (**c**) Power and between-cluster coefficient of variation (km) in the proportion not virally suppressed within matched pairs for different scenarios for realistic, optimistic and pessimistic hypothesised intervention effects with 7 pairs of matched pairs and 200 women per cluster (5 % level of significance)

We enrolled 14 clusters in seven matched pairs in both the baseline and final RDS surveys, aiming to recruit 200 FSW at each site. We used the approach of Hayes and Bennett [8] to determine an appropriate sample size. RDS estimates can have high variance [9, 10], which translates to greater variation between cluster prevalence estimates in our analysis. Given the estimated prevalence of HIV and access to care at baseline, our estimate of intervention effect and the 14 clusters of approximately 200 FSW per site, we calculated that the matched-pair coefficient of variation could be up to k_m_ = 0.2, to achieve 80 % power (Cowan F: Antiretrovirals for HIV prevention and treatment among Zimbabwean sex workers, unpublished) (Fig. 2).

### Additional trial components

Process evaluation will include both the qualitative and quantitative assessment of i) whether intervention activities were conducted as scheduled; ii) ‘sex worker friendliness’ toward outreach clinics; and iii) levels of FSW participation in the intervention. We do not describe this data collection in any further detail in this paper. We will also determine the cost of the intervention components and the cost–effectiveness of the enhanced intervention, taking into account the potential reduction in transmissions via transactional sex due to the reduction in the proportion of FSW who are infectious. For this, we will use an existing individual-based dynamic stochastic model to predict the potential impact of this intervention on HIV incidence in the population of Zimbabwe. Again, these methods are not discussed further here.

### Inclusion criteria and methods for data collection

Primary outcome assessment will be undertaken among women currently working as a FSW (exchanged sex for money in the past 30 days), aged 18 or older, and living or working in the study cluster for at least 6 months. We will use RDS to recruit at each site because it is practically impossible to assemble a sampling frame of the intended target population [11, 12]. At each site, we will first conduct 2 to 3 days of geographic and social mapping, including informal discussions with trained peer educators, healthcare staff, and community informants. This work informs specific criteria for ‘seed’ women to ensure that all sub-populations within the site’s sex worker population are represented and helps determine how many of these seeds should be selected. Approximately six to eight purposefully selected women (the ‘seeds’) in each site will be interviewed and given two recruitment coupons to pass on to their sex worker peers. When the women receiving the coupons attend for the interview (‘recruits’), they are also given two coupons to give out to FSW in that location. In all 14 sites, five iterations of this process (‘waves’) will be performed during both the baseline and endline surveys. Survey data will be collected directly on electronic tablet computers, and the data uploaded to a central database at regular intervals.

### Sample collection and laboratory analysis

All women will have a finger prick blood sample (DBS) collected for the detection of HIV antibody with an AniLabsytems EIA kit (AniLabsystems Ltd, OyToilette 3, FIN-01720, Finland). Blood samples will be air-dried onto filter papers and stored at room temperature until being transported biweekly to the Flow Cytometry Laboratory in Harare. If HIV antibody is detected, then the sample will be retested for HIV viral load using NucliSENS EasyQ HIV-1 v2.0 (bioMérieux, France), both to confirm the HIV positive status and to quantify the viral load (NucliSENS EasyQ HIV-1 v2.0 is approved by the United States Food and Drug Administration, FDA, for use on dried blood spot specimens). For those samples with a positive HIV antibody test using Anilab EIA but an undetectable viral load, a second confirmatory ELISA will be performed (Enzygnost Anti-HIV 1/2 Plus ELISA, Germany). During the baseline survey at the two trial sites, plasma samples will be collected in addition to DBS and tested in parallel using NucliSENS EasyQ HIV-1 v2.0, to permit validation of the use of DBS for viral load quantification in our hands [13]. Informed consent will be obtained from all participants prior to conducting the interview and collecting blood samples.

### Ethics and regulatory approvals

Ethical approval has been received for the trial from the following institutions: the Research Council of Zimbabwe, the Medical Research Council of Zimbabwe, University College London, London School of Hygiene and Tropical Medicine and RTI International. The trial was registered at Pan African Clinical Trials Registry (PACTR201312000722390) on 9 December 2013.

### Design features requiring special consideration in reporting and analysis

Our use of RDS to recruit participants creates several issues for analysis and reporting. We seek to apply the principles and practices of the CONSORT approach for cluster randomised trials, which maximise transparency in reporting [14]. However, some aspects of our design pose challenges in this respect. First, since no sample frame exists from which the research population has been systematically sampled, it is not possible to describe individual non-participation rates in the outcome surveys or to analyse whether these differ by study arm. This step is an important component in the reporting of trial profiles. We describe below the statistics that will be possible to report and consider the strengths and limitations of these. Second, analysing the potential for sampling bias in RDS-based samples from within-study clusters will be an important component of our analysis. Of particular importance for our trial will be to assess whether there is evidence that the operationalization of RDS has introduced systematic participant recruitment bias that differs by cluster, cluster pair, or trial arm. In the case of endline surveys, we will need to investigate whether recruitment patterns through RDS change over time because of either the previous application of RDS in the study clusters, or because of an effect of the intervention in the intervention arm. The intervention seeks to strengthen the social support and increase communication within the social networks of sex workers within each cluster. This intervention might plausibly also influence patterns of peer recruitment to the RDS-survey, and relevant statistics should be reported transparently within our analysis.

Finally, we must select a primary analysis approach suitable for the structure of our data. As described below, since we are conducting a cluster randomised trial with a relatively small number of clusters, we will use a cluster-summaries approach. Our primary analysis will be based on the two-stage approach of Hayes and Moulton [15] adapted for use with RDS. We will consider an unadjusted analysis as a sensitivity analysis. We describe our preferred approach in the next section, and consider the strengths and limitations in relation to alternatives in the discussion.

### Statistical analysis plan

#### Analysis principles

We hypothesise that after 2 years, a lower proportion of FSW from the study clusters, which have been randomised to receive the combination SAPPH-IRe intervention, will have an HIV viral load ≥ 1000 copies/ml than FSW from comparison clusters. Our analysis will be based on an intention-to-treat principle in that the primary analysis will compare FSW recruited in RDS surveys in each community without considering direct contact with the intervention components. Analysis will be appropriate for the matched-cluster-pairs design of the study. Data from individual FSW will be summarised for each cluster as described below, and we will express the intervention effect in the form of a prevalence ratio and show associated 95 % confidence intervals. We will interpret there to be strong evidence against the null hypothesis of no intervention effect if the 95 % confidence interval of the prevalence ratio excludes 1.

Our estimation of cluster summaries will need to take account of the RDS methodology used to recruit participants. A range of literature describes approaches to handling data from RDS surveys. Our approach will be based on the RDS-2 methodology developed by Volz and Heckathorn [16], which has been found to be less biased than the earlier Salganik-Heckathorn estimator [9, 17–19]. RDS-2 conceptualises RDS recruitment as a random walk sampling process throughout the entire social network of FSW at each site. Under this assumption, the probability of recruitment of any one non-seed FSW to the research sample is proportional to their number of FSW social contacts, or ‘degree’ in network terminology [16]. Consequently, FSW with a higher ‘degree’ have a greater probability of appearing in the final research sample than those with a lower degree. In generating cluster-summaries, we will therefore conduct a weighted-analysis in which individuals are weighted with proportion to 1/self-reported out-degree. The question we will use to estimate network degree is ‘the number of sex workers a participant reported knowing who were at least 18 years old, lived at the site, and who the participant would consider recruiting to the study’.

### Study profile

We will describe the number of clusters recruited to each arm of the study at baseline and document the drop-out of any full study clusters during the trial period. Cluster drop-out is not expected but might occur if political or community acceptance for the research protocol is compromised during the trial duration.

For each arm of the study, we will describe, at both baseline and at the end of the follow-up, the range and mean size of the sample recruited through the RDS surveys in each site. We will produce participant recruitment trees to describe the RDS recruitment process in every cluster. Because it is participants who approach other new potential participants, it is not possible for us to directly measure the refusal rate. We will describe by arm and at baseline and endline, the range and cluster-mean of the number of women who do not forward recruit two participants. At endline, we will ask women how many FSW they tried but failed to recruit and their understanding of the reasons for their refusal. For each arm, the range and mean of participants in each cluster with missing data for the primary outcome will also be documented. These data will be used to construct a trial profile diagram in line with CONSORT principles, but adapted for our specific situation.

### RDS diagnostics

The random walk model upon which RDS-2 estimation is based makes a number of assumptions about the sampling process. These include that participants are able to accurately report their network size and that they recruit randomly from within this network; that seed characteristics do not bias the final estimates; that the whole social network of female sex workers is connected at each site; and that social ties are reciprocated (recruitees also know their recruiters). The model also assumes with-replacement sampling – that is, that participants can be recruited more than once, which is not the case in practice. However, the extent to which some of these assumptions might be biasing study findings can be tested using information collected in the survey and from a brief follow-up interview when participants return to collect their incentives for recruiting others. We will conduct a set of recommended RDS diagnostics for each site, as recommended by Gile et al. [20], to assess the extent to which the actual sampling process differed from the model. We will report our findings according to emerging STROBE guidelines for the reporting of RDS surveys [21].

RDS studies have typically assessed the possible impact of biases on an estimated proportion through simulation of a social network and/or of the RDS process that runs over it [17, 22, 23], through there are a few examples where biases could be empirically tested [9, 24]. For our study, we are primarily interested in assessing whether deviation from the assumed recruitment process might be differential by intervention arm and thus differentially biasing site estimates and study findings. We therefore investigate assumptions as follows, by study arm.

First, for each site and categorised by arm, we will examine graphically the extent to which the cumulative estimates for our primary and secondary outcomes stabilise from the initial seed characteristics over successive waves of recruitment [20]. The random walk model requires the assumption that the final estimates are no longer influenced by initial seed characteristics. Secondly, we will use the same graphical method to assess whether the estimates for each seed converge. If seed-specific estimates remain different from each other by the final sample wave, this observation might suggest that the population is split or extremely clustered into separate sub-groups rather than fully connected as the random walk model assumes [20].

We will also assess whether deviation from random recruitment varies by arm [25]. When we ask participants to estimate their network size, we will also ask them to describe the characteristics of these FSW, including the proportion who are under 25 years, who engage in different types of sex work and other characteristics to judge whether recruitment from among each woman’s pool of potential recruitees is related to any of these factors. We will also ask participants to describe their relationship to their recruiter and recruitees to confirm reciprocity (that both women did know each other previously as the model assumes). This will enable us to assess the random recruitment assumption by comparing the recruiter-recruitee characteristics to other women in the group of potential recruitees [26].

RDS-2 estimates have been found to be sensitive to errors in reported degree, particularly in participants with low degrees whose status has a higher weight [27]. When participants return to collect incentives, we will ask them to estimate the size of their network a second time, and we will calculate test-retest reliability of this estimate. RDS also assumes that all social contacts are reciprocated, so we will investigate the extent to which this is true and the extent to which recruitment might vary by relationship characteristics [28] by asking participants about their relationship with their recruiter.

If the community mobilisation and adherence support activities change the structure and composition of the FSW social networks in the intervention sites, possibly, the RDS sampling process that runs over these networks might be differentially biased by the trial arm. RDS surveys have been used to characterise the underlying social network [22, 29]. To investigate whether unobservable biases might have occurred, we will examine the measures that describe the character of the FSW social network at each site by: 1) comparing changes in mean and median degree reported at baseline and at follow-up in intervention versus usual care sites; 2) comparing the levels of homophily (similarity) between FSW and their personal networks, and between recruiters/recruitees; 3) comparing changes in reported social cohesion and the proportion of sex workers reporting good relations with other sex workers; and 4) examining whether the conditions of recruitment (place of recruitment, relationship between recruiter/recruitee, and motivation for recruitment) differ between arms.

### Assessment of baseline balance and description of participants

We will calculate cluster-summaries for key sociodemographic characteristics of the sample recruited through RDS at both baseline and at endline, and report the cluster-mean and range for these, stratified by study arm. At baseline, we also estimate the level of inter-cluster variability in the primary outcome variable expressed in the form of the inter-cluster-pair coefficient of variation, known as k_m_ [8].

### Operationalising the primary outcome variable

All consenting women recruited to the RDS surveys will be tested for HIV antibody (laboratory analysis details below). Bloodspot samples from all women with detectable HIV antibody are then tested for viral load. For the analysis, those women who have ≥ 1000 copies/ml detected were categorised as having a detectable viral load. The primary outcome variable will be calculated as:

$$ \begin{array}{l} = \mathrm{Number}\ \mathrm{of}\ \mathrm{women}\ \mathrm{with}\ ``\mathrm{detectable}"\ \mathrm{H}\mathrm{I}\mathrm{V}\ \mathrm{an}\mathrm{tibody}\ \mathrm{an}\mathrm{d}\ \mathrm{d}\mathrm{etectable}\ \mathrm{viral}\ \mathrm{load}/\\ {}\mathrm{All}\ \mathrm{women}\ \mathrm{who}\ \mathrm{had}\ \mathrm{an}\ \mathrm{H}\mathrm{I}\mathrm{V}\ \mathrm{an}\mathrm{tibody}\ \mathrm{test}\ \mathrm{performed}\end{array} $$

A range of secondary endpoints are described in the study protocol but are not discussed in detail here.

### Primary outcome analysis strategy

Our primary analysis will be a comparison of RDS-2 weighted, adjusted, endline cluster summaries of the primary outcome, adjusting for cluster summaries at baseline. We will then fit a linear regression model on the endline summaries, adjusting for the baseline cluster summaries and a dummy variable for the pair. We will calculate and report prevalence ratios and risk differences.

To address the possibility of confounding by individual and cluster-level variables from chance imbalances, we will conduct an adjusted analysis. This analysis will use the ‘two step’ method from Hayes and Moulton to adjust for differences in covariates at endline [15]. Potential confounders will be included that could affect the outcome but that are very unlikely to be on the causal pathway: education and age.

The two-step method will be applied to the endline cluster summaries. Step 1: For each site a logistic regression model will be fitted with the primary outcome as the dependent variable, and education, age, and pair as independent variables. This model will be used to ‘predict’ outcomes for each woman. To account for the sampling design, we will multiply the predicted values by the inverse of the degree (RDS-2), normalise for the cluster and calculate cluster summaries. Step 2: We will then divide the cluster summaries used in the unadjusted analysis (that is, accounting for RDS only) by the adjusted cluster summaries from Step 1 to generate ‘residuals’. We will calculate the effects using the regression model described above with the residuals as the dependent variable.

### Sensitivity analyses

Finally, we will produce a range of sensitivity analyses that investigate how robust our primary effect estimate is to a range of different assumptions. We will first recalculate the primary effect estimate using cluster-summaries that are not adjusted for the RDS-2 methodology. To investigate the sensitivity of the RDS-2 weighted findings to participants’ estimates of their network size, we will recalculate the estimated effect using the higher reported degree of the follow-up and main survey if these differ. Finally, the random walk model assumes with-replacement sampling, while participants, in fact, cannot participate more than once in the RDS-2 survey. The RDS-2 estimator has been found to be biased when the sampling fraction is large and when there is a large difference in the network sizes of those with and without the outcome of interest (‘differential activity’) [17]. Another RDS estimator, the ‘Successive Sampling’ estimator (‘RDS-SS’), has been designed to avoid the without-replacement assumption but requires estimates of the population size of sex workers [30]. We will conduct the analysis using this estimator as a sensitivity analysis for a range of possible population sizes [31] and assess whether our study findings differ using these estimates.

## Discussion

Pragmatic trials of the effectiveness of complex, combination intervention packages are growing in importance, but pose challenges for design, analysis and reporting. In comparison to individual phase II or III trials of biomedical interventions, it is more important in pragmatic trials that the study sample is representative of the target population for public health impact. However, where no sampling frame exists for the target population, as it is the case for FSW, novel methods are required for recruitment. We describe the design and analysis plan for a pragmatic trial being conducted in Zimbabwe on a combination HIV prevention and treatment intervention for FSW, in which research participants contributing to primary endpoint analysis will be recruited through respondent-driven sampling surveys. We describe the analysis steps we will go through to report the profile of the trial, assess risk of selection bias and calculate the primary treatment effect given this unusual design.

Our trial design and analysis strategy has many strengths. Our clustered, rather than individually randomised, design captures the effects of the intervention on the proportion of all FSW at the site with a detectable HIV viral load. This outcome measure is a good indicator of the potential of the intervention to reduce HIV transmission, as well as the impact of treatment at the individual level to guarantee the best prognosis. Unlike many earlier HIV prevention interventions [32], our trial design accounts for the clustered nature of our data.

Studies of sex workers employing convenience sampling severely limit the generalisability of their findings to routine practice. In many cases, it is likely that those members of a hidden population who are most reachable to researchers are also likely to be most reachable by service programmes. Each of the methods proposed to obtain near-to-representative samples of hidden populations, including venue-based methods such as ‘time location sampling', makes assumptions about that population, and there is no one agreed-upon best method for all cases [12]. We have previously found RDS recruitment among FSW in Zimbabwe to be feasible and did not detect major biases [33].

We recognise that there are potential limitations and threats to the research. The need to minimise contamination between communities has meant that we have only 14 clusters, which could make finding an effect challenging. While RDS weighting is intended to reduce bias in chain referral sampling, simulation and empirical studies have shown that variance around the weighted point estimates for each cluster could be very high [9, 10]. In our study, this variance will be reflected in the between-cluster differences. Our power calculations have accounted for a between-matched-pair coefficient of up to 0.19, and at baseline we calculated k_m_ to be 0.18 (Cowan F, et al. HIV care cascade among female sex workers in Zimbabwe: baseline results of the SAPPH-IRe Trial, submitted). Additionally, we have sought to improve power by matching sites and adjusting our final analysis by baseline age and education characteristics.

While our aim of assessing the intervention under routine conditions makes recruiting a representative sample of FSW important, this is challenging when we lack a sample frame. We have sought to address this problem by using RDS, but we recognise that this is not a panacea in delivering unbiased representative estimates. Often RDS is implemented variably, limiting comparability. A strength of our study is the identical protocol implemented at each study site. We are aware of one other application of RDS in a cluster randomised trial, but to our knowledge no trials have yet been completed [34, 35]. We have described a range of potential biases that can arise from differences in the assumed and real sampling process under RDS. We have adapted our surveys to collect the information required to signal whether any of these biases could be present, and we have planned sensitivity analyses to assess their impact on our study findings.

Potentially most seriously, it is plausible that these biases could be differential by study arm, given that the community mobilisation component of the intervention could alter the social networks of FSW along which our endline recruitment will proceed. It is common for interventions aiming to reach socially marginalised and criminalised populations to employ methods that target social networks and to encourage collective mobilisation [36–38]. Because RDS and other approaches using social networks are also recommended for use in recruiting participants for research, it is likely that other evaluation efforts will face similar challenges with differential biases derived from intervention changes in those network characteristics. Our approaches to assessing the potential for this bias will therefore be applicable more broadly to research in reaching those populations most affected by HIV.

Trials to identify new biomedical interventions are necessary, but not sufficient, to provide an evidence base to help support global health gains. The complexities of ensuring real public health gains through effective, equitable implementation of these tools requires that we also expend significant energies on research [39]. Our research is in this latter mode: we would like to see a range of pragmatic studies conducted that help incrementally build an evidence base to support maximum impact of HIV prevention technologies among groups such as FSW. Our contribution advances these efforts in providing a description of how such a trial can be designed in a hard-to-reach population severely affected by HIV, and key points to consider in analysis and in translating guidance on cluster RCTs in the context of using RDS for recruitment.

## Trial Status

The trial completed baseline data collection from sites in December 2013, and endline data collection will take place April and May 2016.

## Abbreviations

ART: antiretroviral therapy: treatment for HIV infection
ARV: antiretrovirals: drugs used for the treatment of HIV infection
CONSORT: Consolidated Standards of Reporting Trials: initiative with associated guidelines aimed at strengthening the reporting of randomised controlled trials
DFID: Department for International Development, United Kingdom government
DBS: dried blood spot: finger prick blood samples collected from participants and dried on filter paper for HIV antibody and viral load testing
ELISA: enzyme-linked immunosorbent assay: used to screen blood for presence of antibodies to HIV, for the purposes of testing for HIV infection
FDA: Food and Drug Administration, United States government
FSW: female sex workers
HIV: human immunodeficiency virus
PrEP: pre-exposure prophylaxis: antiretroviral drugs taken by HIV negative individuals to prevent infection with HIV
RCT: randomised controlled trial
RDS: respondent-driven sampling: a form of chain referral sampling used to recruit participants
RDS-2: An estimation procedure applied to prevalence estimates from data collected via respondent driven sampling to account for sampling design
RDS-SS: respondent-driven Sampling – successive sampling estimator: an alternative respondent driven sampling estimator
SAPPH-IRe: Sisters Antiretroviral Programme for Prevention of HIV – an Integrated Response: name of the trial
STROBE: STrengthening the Reporting of OBservational studies in Epidemiology: initiative with associated guidelines and checklists for the reporting of observational studies
Swedish Sida: Swedish Development Agency
UNFPA: United Nations Population Fund
WHO: World Health Organization
